# Argon plasma modification promotes adipose derived stem cells osteogenic and chondrogenic differentiation on nanocomposite polyurethane scaffolds; implications for skeletal tissue engineering

**DOI:** 10.1016/j.msec.2019.110085

**Published:** 2019-12

**Authors:** Michelle F. Griffin, Amel Ibrahim, Alexander M. Seifalian, Peter E.M. Butler, Deepak M. Kalaskar, Patrizia Ferretti

**Affiliations:** a UCL Centre for Nanotechnology and Regenerative Medicine, Division of Surgery & Interventional Science, University College London, London, United Kingdom; b Royal Free London NHS Foundation Trust Hospital, London, United Kingdom; c Stem Cells and Regenerative Medicine Section, UCL Institute of Child Health, University College London, London WC1N 1EH, United Kingdom

**Keywords:** Adipose stem cells, Osteogenesis, Chondrogensis, Argon, Plasma surface modification

## Abstract

Bone and cartilage craniofacial defects due to trauma or congenital deformities pose a difficult problem for reconstructive surgeons. Human adipose stem cells (ADSCs) can differentiate into bone and cartilage and together with suitable scaffolds could provide a promising system for skeletal tissue engineering. It has been suggested that nanomaterials can direct cell behavior depending on their surface nanotopographies. Thus, this study examined whether by altering a nanoscaffold surface using radiofrequency to excite gases, argon (Ar), nitrogen (N_2_) and oxygen (O_2_) with a single step technique, we could enhance the osteogenic and chondrogenic potential of ADSCs. At 24 h, Ar modification promoted the highest increase in ADSCs adhesion as indicated by upregulation of vinculin and focal adhesion kinase (FAK) expression compared to O_2_ and N_2_ scaffolds. Furthermore, ADSCs on Ar-modified nanocomposite polymer POSS-PCU scaffolds upregulated expression of bone markers, alkaline phosphatase, collagen I and osteocalcin after 3 weeks. Cartilage markers, aggrecan and collagen II, were also upregulated on Ar-modified scaffolds at the mRNA and protein level. Finally, all plasma treated scaffolds supported tissue ingrowth and angiogenesis after grafting onto the chick chorioallantoic membrane. Ar promoted greater expression of vascular endothelial growth factor and laminin *in ovo* compared to O_2_ and N_2_ scaffolds as shown by immunohistochemistry. This study provides an important understanding into which surface chemistries best support the osteogenic and chondrogenic differentiation of ADSCs that could be harnessed for regenerative skeletal applications. Argon surface modification is a simple tool that can promote ADSC skeletal differentiation that is easily amenable to translation into clinical practice.

## Highlights

• Bone and cartilage craniofacial defects due to trauma or congenital deformities pose a challenging problem for reconstructive surgeons.
• Nanomaterials can direct adipose derived stem cell (ADSC) differentiation depending on their surface nanotopographies.
• This study demonstrates that Argon surface modification improve the chondrogenesis and osteogenesis of ADSCs.
• Argon surface modification is a tool that can upregulate ADSC skeletal differentiation and is amenable to translation into clinical practice.

## 1 Introduction

Bone and cartilage craniofacial defects due to burns, trauma or congenital deformities pose a challenging problem for reconstructive surgeons. Bone defects of the mandible and maxilla are very common; they can be caused by several pathologies including cancer resection, trauma, and congenital deformities [1]. In addition, 1 in 6000, children worldwide are born with a small or little ear, a condition called microtia [1]. Currently reconstructive surgeons utilize bone and cartilage grafts harvested from elsewhere in the body (*e.g.* skull, ribs) to reconstruct the defect impeding donor site morbidity and needing to overcome the limitation of free bone tissue [1]. Several natural and synthetic biomaterials have been investigated to serve as scaffolds to encourage new bone or cartilage in-growth and overcome the harvesting of autologous tissue to restore bone or cartilage defects [1].

The field of nanotechnology has led to the development of materials, which mimic the nanoscale dimensions of the native extracellular matrix to improve cell-biomaterial interaction. Nanomaterials can direct cell behavior due to the surface nanotopographies and incorporation of specific nanoparticles. We have previously investigated a non-biodegradable nanocomposite polymer, which integrates the polyhedral oligomeric silsesquioxanes (POSS, (RSiO3/2)n) nanoparticle with the polyurethane back-bone poly(carbonate-urea)urethane (PCU), to create tissues to restore damaged organs [[2], [3], [4], [5]]. The polymer has shown good biocompatibility and haemocompatibility with multiple cell lines including fibroblasts, endothelial cells, mesenchymal stem cells and thus is a good candidate to be investigated as a biomaterial to restore facial defects [[2], [3], [4], [5]].

Engraftment and retention of biomaterials is enhanced by the inclusion of suitable cells. Stem cells derived from adult or paediatric adipose tissue (ADSCs) are known to be valuable autologous cell sources for regenerative research due to their simple isolation method, ease of expansion *vitro* and multi-lineage differentiation capacity [1,6]. Furthermore, they are readily available in the reconstructive surgical setting and thus a suitable stem cell source for surgical facial reconstructive applications [1,6]. We have previously shown the ability of POSS-PCU to support both osteogenic and chondrogenic differentiation of ADSCs as well as the ability to populate the scaffolds and support blood vessel formation *in ovo* [2].

Interactions at the material-cell interface can influence stem cell proliferation and differentiation [3]. Several techniques have been used to improve stem cell differentiation towards bone and cartilage including modifications to the scaffold architecture and composition or the scaffold surface [8]. Scaffold surface modifications include modifying the surface topography including the wettability, stiffness, roughness to direct stem cell behaviour [9]. Alternatively the scaffold surface can be modified with growth factors to provide appropriate biosignals [8]. However, the ideal surface technique to modulate osteogenesis and chondrogenesis is still under debate. The plasma surface modification (PSM) tool is a simple surface-modifying technique which can change the surface topography and chemistry to enhance cell adhesion to biomaterials and promote desired cell responses [3,7]. Radiofrequency plasma is the most commonly used form of PSM for modifying the surface of biomaterials, which entails passing an electric current though a gas [7]. During the process the polymer surface is bombarded with electron and ions from the plasma phase, which induces an etching effect [7]. PSM is either a single or two-step process [7]. We have previously reported that activating the POSS-PCU surface with gases and then in a second step further depositing a layer of amine or carboxyl functional chemical groups can enhance the osteogenic and chrondrogenic differentiation of ADSCs respectively [10]. However, this is a two-stage technique and thus our aim was to improve this technique by using a single step for activating the surface without the need for deposition of amine or carboxyl groups to make the process simpler and allow for easy translation to clinical practice.

To this purpose, we evaluated the effect of PSM with the three commonly used gases, argon (Ar), nitrogen (N_2_) and oxygen (O_2_), which activate the surface to understand their effect on ADSC osteogenic and chondrogenic differentiation. We have recently fully characterized the effect of N_2_, Ar and O_2_ on polyurethane scaffolds including the surface topographical changes that occur [11]. All the plasma gases reversed the hydrophobicity effectively after 5 min of treatment and allowed for cell adhesion, formation of extracellular matrix and tissue formation and angiogenesis *in vivo* [11]. The three gases have varying effects on the polyurethane scaffolds surface properties but do not influence bulk mechanical properties. All three gases create a hydrophilic polyurethane scaffold as shown by their contact angle measurements [11]. However, O_2_ treatment increases the surface roughness of the polyurethane scaffolds compared to N_2_ and Ar modifications as well as the surface elastic modulus, as demonstrated by atomic force microscopy [11]. Surface chemistry analysis reveals that Ar causes an increase in oxygen content and a reduction in carbon on the surface, but to a lesser degree than O_2_ treatment [11]. The N_2_ treatment does not alter polymer surface chemistry significantly [11]. Overall O_2_ treatment leads to widespread changes in interfacial properties, However, Ar causes less prevalent changes and N_2_ causes the least effect to the polymer surface (Supplementary Fig. 1) [11]. Thus given the different effects of the three gases on the material surface properties, we aimed to understand which PSM is optimal for ASDC proliferation, ostogenic and chondrogenic differentiation.

The results reported here demonstrate that ADSC proliferation and osteogenic and chondrogenic differentiation is enhanced by Ar plasma surface modification for 5 min to a greater degree than O_2_ and N_2_ and allows for vascularization of scaffolds *in vivo*. This study provides an important understanding into which surface chemistries best support the osteogenic and chondrogenic differentiation of ADSCs that could be harnessed for regenerative skeletal applications.

## 2 Materials and methods

### 2.1 Three-dimensional (3D) scaffold manufacture

The POSS-PCU scaffolds were manufactured, as described previously [9]. The polymer was fabricated as a 3D-scaffold using the phase-separation (coagulation)/porogen technique as previously reported [9]. Sodium chloride (NaCl, particle size 200–250 μm) was mixed with POSS-PCU in dimethylacetamide (DMAC) in a 3:7 ratio. The final polymer mixture poured onto steel moulds and then washed in deionized water for 3–5 days to remove the NaCl and DMAC out of the scaffolds. For experiments, 16 mm diameter disks were cut and sterilised using a standard autoclave protocol.

### 2.2 Plasma surface modification (PSM) of 3D scaffolds

A radio frequency plasma generator was used to perform PSM at 40 KHz at 100 W for 5 min as previously reported [11] to ensure the scaffolds received Argon (Ar) nitrogen (N_2_) and oxygen (O_2_) surface modification.

### 2.3 Human adipose stem cell (ADSC) culture on 3D scaffolds

ADSCs were isolated from discarded adipose fat harvested from consented patients under ethical agreement (North Scotland ethical review board 10/S0802/20) and following to the protocol by Naderi et al. [12]. For the *in vitro* analysis, passage 2–4 ADSCs were seeded on the polymer disks.

### 2.4 Adipose stem cell differentiation protocol on 3D scaffolds

Scaffolds were placed at the bottom of the 24 well plate, incubated overnight with ADSC culture medium, and then 10 [5] ADSCs seeded onto each scaffold in fresh medium (day 1). Once confluent (usually on day 3), ADSCs were differentiated according to previous chondrogenic and osteogenic protocols [2]. Following three weeks of chondrogenic or osteogenic differentiation the scaffolds were either fixed in 4% paraformaldehyde for immunocytochemistry or differentiation staining or processed for RNA extraction for RT-qPCR analysis. Analysis of the cell supernatant was also performed for ELISA analysis over the three weeks to evaluate the proteins secreted. Chondrogenic and osteogenic differentiation were assessed by alcian blue and alizarin red staining, respectively, as previously reported [2].

### 2.5 Morphology staining

Actin staining was used to stain the ADSC morphology as previously described [8]. For analysis 15,000 ADSCs were seeded and then stained with Rhodamine-conjugated phalloidin (ThermoFisher Scientific, UK) for actin and with DAPI (4′,6-diamidino-2-phenylindole, 1:500) to stain the nuclei. The cells were analysed using ImageJ Software 1.48 V for cell circularity and cell area. A total of 30 cells were analysed from 6 scaffolds (n = 6).

### 2.6 Live dead assay

After culturing the cells for 6 h on plasma-modified or control (unmodified) scaffolds, ADSC survival was investigated using a live/dead two-colour assay (LIVE/DEAD™ Viability/Cytotoxicity kit Invitrogen). The cells were incubated with the fluorescent dyes for 45 min and analysed by confocal laser scanning microscopy (LSM, 710, Zeiss).

### 2.7 Metabolic activity - Alamar blue™ assay

ADSC cytotoxicity and viability was assessed with Alamar blue™ (Life Technologies, UK) on days 1, 2, 4, 7, 14 and 21 as previously described [2] (n = 6).

### 2.8 Analysis of cell proliferation using DNA quantification

To assess ADSC cells proliferation a Fluorescence Hoechst DNA Quantification Kit at days 1, 2, 4, 7 and 14 (n = 6) as previously described [8].

### 2.9 Quantification of secreted proteins by ADSCs

The elastin (Biocolour Fastin Elastin Assay) and osteocalcin (R&D) secretion by the ADSCs into the culture medium was assessed at 14- and 21-days using ELISA analysis. Collagen secretion was analysed using Pico Sirius Red (PSR) method and hydroxyproline quantification as previously described [13].

### 2.10 Alkaline phosphatase assay

The colorimetric alkaline phosphatase (ALP) assay kit (Abcam) was used to assess ALP activity in ADSCs after 14 and 21 days (n = 6).

### 2.11 Analysis of extracellular matrix (ECM) and adhesion proteins using immunocytochemistry

After 21 days in culture, the scaffolds were washed in PBS and fixed in 4% PFA overnight at 4 °C, as previously described [9]. In summary, scaffolds were embedded in OCT compound and cryosectioned (40 μm thick). Following permeabilisation and blocking scaffolds were incubated with primary antibodies diluted in blocking solution overnight at 4 °C as described previously including ALP for osteogenesis, aggrecan for chondrogenesis and vinculin for adhesion [10]. Following incubation with secondary antibody for 2 h and the cell nuclei staining with Hoechst 33258 (2.5 μg/ml final concentration), scaffolds were imaged using a confocal laser-scanning microscope (LSM 710, Zeiss).

### 2.12 RT-qPCR analysis of ECM and adhesion proteins

In brief, the mRNA expression of chondrogenic and osteogenic differentiation was assessed using RT-qPCR. In summary, the RNA was extracted from the scaffolds at day 21 using Tri-Reagent (Life Technologies) [9,10]. Primer sequences and annealing temperatures for each set of primers was conducted as described previously [10]. The qPCR was performed with an ABI Prism 7500 sequence detection system (Applied Biosystems) with QuantiTect SYBR Green PCR kit (Qiagen, Hilden, Germany) [9,10].

### 2.13 Chorioallantoic membrane (CAM) grafting

To assess the angiogenesis of the ADSC seeded scaffolds CAM assays were performed as previously described [10]. After 3 days of incubation in a 37 °C incubator eggs were windowed. At 7 days CAMs was scratched and scaffolds seeded with ADSCs were then grafted (n = 6). Following further incubation for 7 days at day 14 the eggs were photographed *in ovo*. Following this, the scaffolds were processed for immunocytochemistry staining as described previously [10] for vascularization markers including vascular endothelial growth factor (VEGF) and laminin.

### 2.14 Statistical analysis

Graph Pad (Prism) was used to conduct the statistical analysis using ANOVA and Tukey HSD post-hoc analysis. A p < 0.05 value was considered statistically significant.

## 3 Results

### 3.1 ADSC adhesion on plasma modified scaffolds

**Fig. 1:**
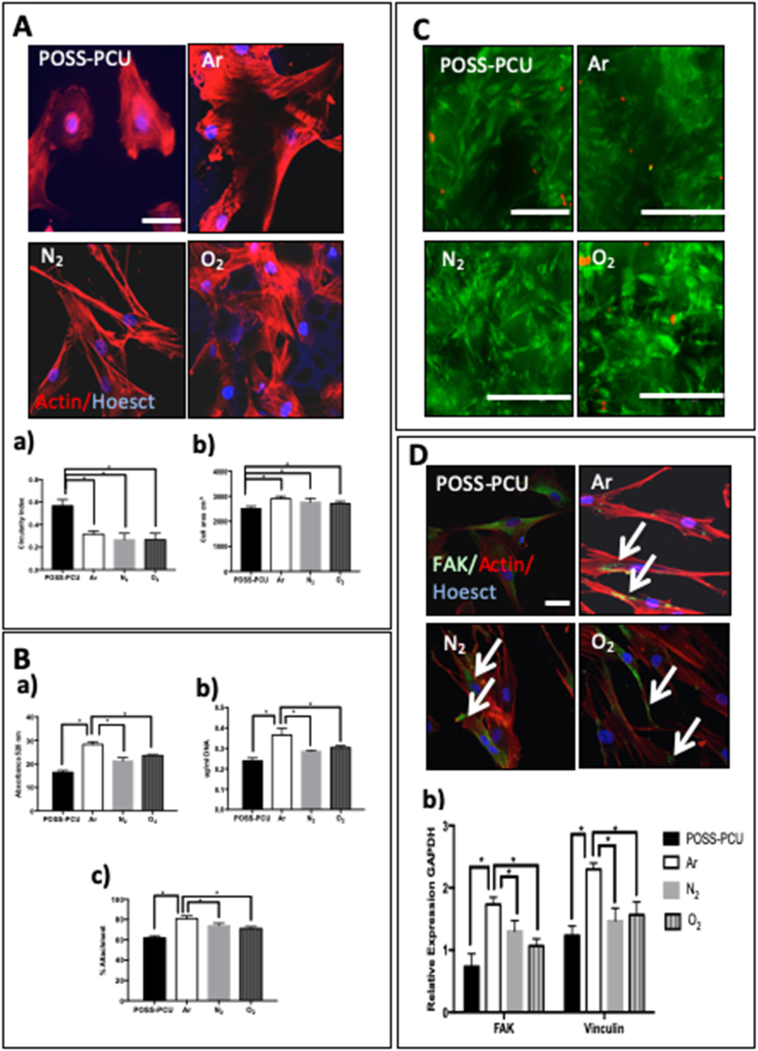
Adhesion, spreading and viability of adipose-derived stem cell (ADSC) on plasma-modified scaffolds. A) Detection of actin by phalloidin staining (red) in ADSCs 6 h after seeding on control and modified POSS-PCU scaffolds. Nuclei are stained with Dapi (blue). Scale Bar: 20 μm. Measure of (a) circularity index and (b) actin-covered area of ADSCs on the different scaffolds at 24 h; note significantly decreased “actin” area and increased circularity index on unmodified scaffolds * p < 0.05.* B) Assessment of cell viability (a), DNA content (b), and percentage cell attachment (c) in ADSC at 24 h. Note that DNA content and cell viability are significantly greater on the Ar-modified scaffolds compared to all other scaffolds (* p < 0.05). C) Live dead assay showed there was minimal dead cells on all scaffolds after 24 h of cell seeding (Live; Green Dead; Red). D) Adhesion of ADSCs on plasma modified scaffolds 24 h after seeding. Double staining for vinculin (green) and actin (red); note expression of vinculin in all scaffolds. Nuclei are stained with Hoechst (blue). POSS-PCU; Unmodified scaffolds, Ar: argon, N_2:_ nitrogen, O_2:_ oxygen. Scale bar: 20 μm a) Expression of focal adhesion kinase (FAK) and vinculin assessed by RT-qPCR. Note greater expression of these transcripts on the plasma modified scaffolds (p < 0.05). (For interpretation of the references to colour in this figure legend, the reader is referred to the web version of this article.)

The biocompatibility of the plasma-modified scaffolds after 5 min of Ar, N_2_ and O_2_ treatment was examined. F-actin was used to compare the ADSCs morphology after 6 h, as cells cannot be easily imaged on POSS-PCU scaffolds. After 6 h the ADSCs showed a more stretched morphology with a similar cell area compared to unmodified scaffolds using actin staining (p < 0.05) (Fig. 1A). After 24 h, a significantly greater number of ADSCs adhered to Ar-modified scaffolds compared to N_2_, O_2_ and unmodified scaffolds as shown by DNA content and Alamar Blue viability assays (p < 0.001) (Fig. 1B). After 6 h live dead staining demonstrated a similar number of live cells after PSM, with very few dead observed (Fig. 1C). Focal adhesion kinase (FAK) encodes a cytosolic tyrosine kinase, which enables cells to agree to their extracellular matrix *via* the formation of focal adhesion complexes (FACs). Similarly, vinculin is a membrane cytoskeletal protein involved in the formation of FACs (Fig. 1D). After 24 h, vinculin and FAK expression was significantly greater on the Ar-modified scaffolds than on O_2_, N_2_ or unmodified scaffolds (Fig. 1D) (p < 0.05).Furthermore, vinculin and actin staining showed the ADSCs had a stretched morphology and expressed vinculin on all PSM scaffolds (Fig. 1D) after 24 h and unmodified scaffolds (Fig. 1D).

### 3.2 Analysis of ADSC proliferation on plasma modified scaffolds

**Fig. 2:**
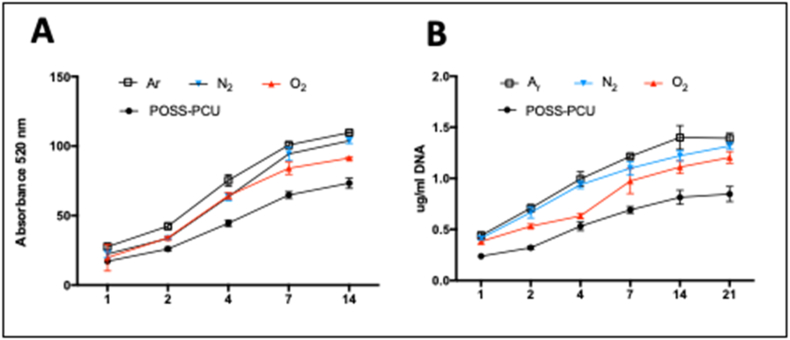
Proliferation of adipose-derived stem cells (ADSCs) on plasma modified scaffolds. Long-term culture of the ADSCs on the plasma modified scaffolds. A) Alamar blue assay and B) DNA assay confirmed the long-term culture of ADSCs on plasma-modified scaffolds over 21 days was the greatest on Ar scaffolds compared to N_2_, O_2_ and unmodified scaffolds (p < 0.05). (For interpretation of the references to colour in this figure legend, the reader is referred to the web version of this article.)

The ADSC proliferation and viability was anlayed over 21 days following surface modification (Fig. 2A, B). The ADSCs viability was significantly higher on modified compared to unmodified scaffolds, but all scaffolds supported cell growth with long-term culture (p < 0.005) (Fig. 2A). Total DNA assay demonstrated PSM scaffolds with Ar enabled greater cell growth over 21 days (p < 0.05) (Fig. 2B).

### 3.3 Osteogenic and chondrogenic differentiation on plasma modified scaffolds

**Fig. 3:**
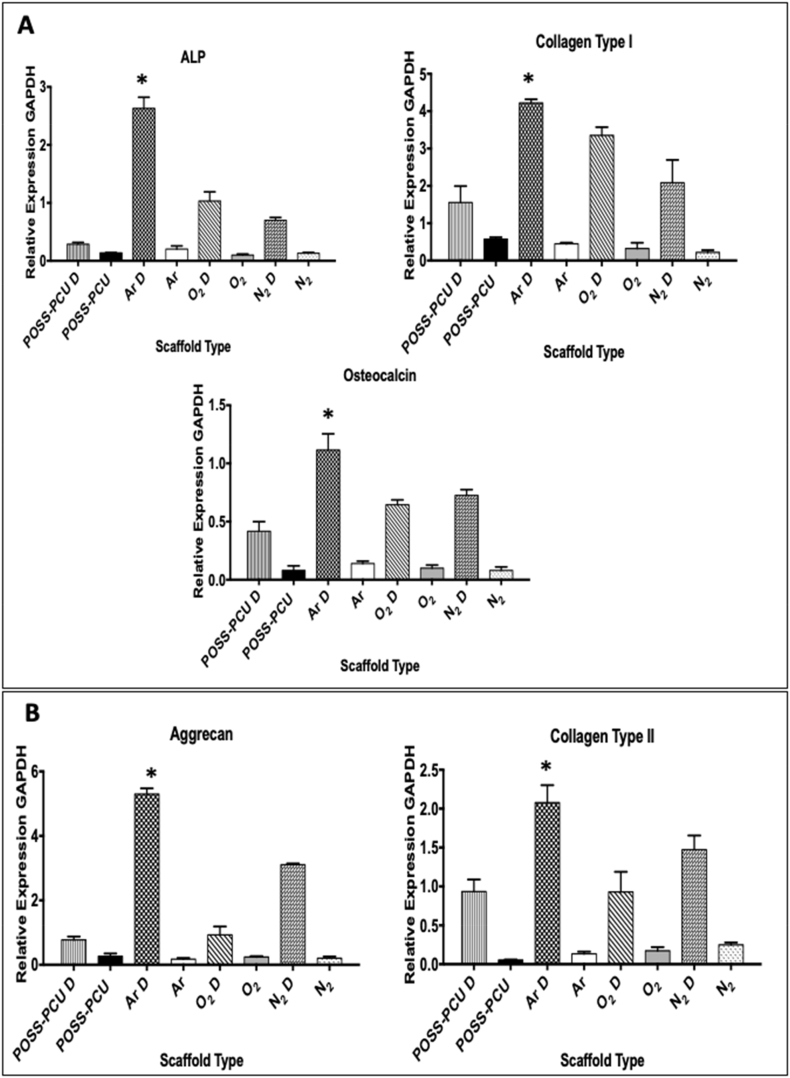
Differentiation of adipose-derived stem cells (ADSCs) on plasma modified scaffolds assessed by RT-qPCR after 21 days of chondrogenic and osteogenic differentiation. A) Expression of alkaline phosphatase (ALP), collagen type I and osteocalcin following ADSC osteogenic differentiation; upon differentiation gene expression increase is greater on Ar modified scaffolds than on the other scaffolds (*p < 0.05). B) Expression of aggrecan and collagen type II following ADSC chondrogenic differentiation (indicated by “D”); upon differentiation both transcripts are significantly higher on the Ar scaffolds than on the other scaffolds. POSS-PCU; Unmodified scaffolds, Ar; Argon, N_2;_ Nitrogen, O_2_; Oxygen.

The extent of ADSC differentiation down the osteogenic and chondrogenic pathways on the modified scaffolds was assessed at 3 weeks. Expression of tissue-specific differentiation markers was determined by RT-qPCR (Fig. 3). The gene expression of osteogenic markers including collagen I, alkaline phosphatase and osteocalcin was also significantly greater on Ar plasma modified scaffold (p < 0.05) (Fig. 3A). Expression of aggrecan and collagen II, markers of chondrogenic differentiation, was up-regulated on all plasma-modified scaffolds, with the greatest on Ar scaffolds compared to all other modified and unmodified scaffolds (p < 0.05) (Fig. 3B). Quantification of the chondroid matrix using Alcian blue staining and osteoid using Alizarin Red Staining also confirmed Ar modification promoted the osteogenic and chondrogenic differentiation of the ADSCs, completing the mRNA studies (Supplementary Fig. 2).

Evaluation of the differentiation at the protein level on the PSM scaffolds was assessed at 3 weeks by immunocytochemistry. Following osteogenic differentiation, greater expression of ALP was observed on all PSM scaffolds compared to control scaffolds, but to a greater degree on the Ar scaffolds (Supplementary Fig. 3). Similarly, expression of the cartilage marker aggrecan, was greater on PSM scaffolds compared to control scaffolds with the strongest staining observed in Ar modified scaffolds (Supplementary Fig. 3). Increased secretion of osteocalcin, total collagen and ALP activity on the Ar scaffolds in osteogenic medium was consistent with enhanced osteogenesis on these scaffolds (Supplementary Fig. 2). The secretion of elastin and glycosaminoglycans by the ADSCs was also greater on the Ar scaffolds, further supporting enhanced chondrogenesis on these scaffolds (Supplementary Fig. 2). No mRNA upregulation was observed in ADSCs without differentiation medium, demonstrating that PSM improves ADSC differentiation, but not able to drive tissue specific differentiation of these cells independently (Fig. 3).

### 3.4 Vascular response to ADSC-POSS-PCU bioscaffolds

**Fig. 4:**
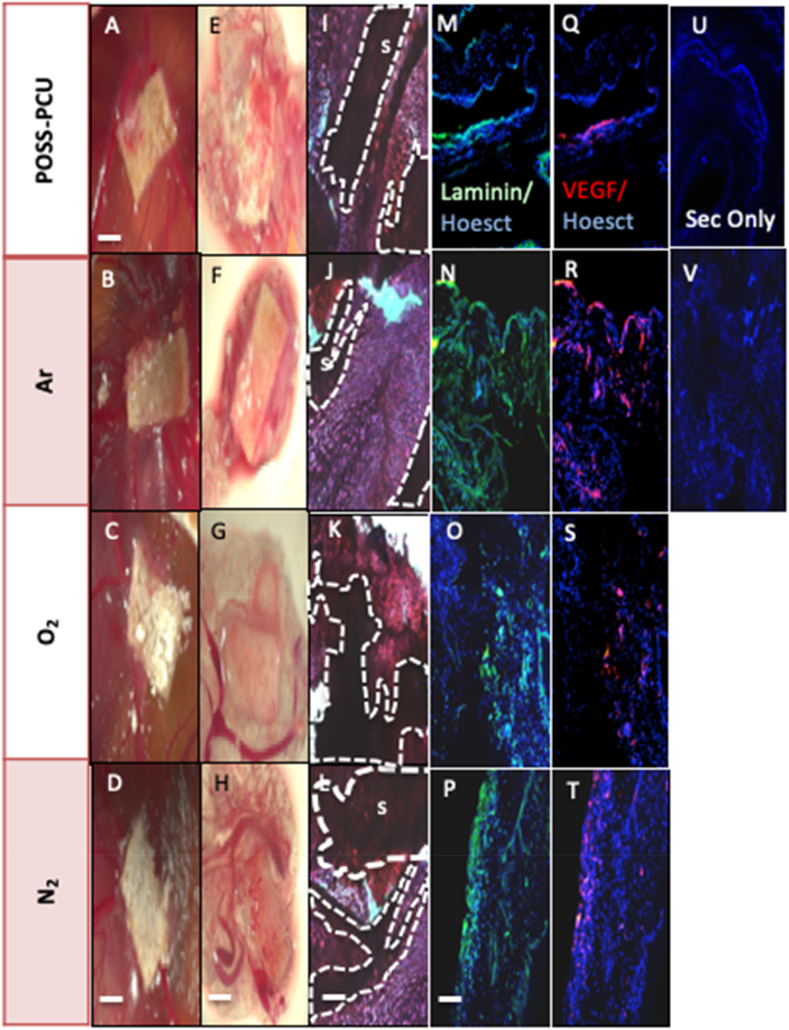
Chorioallantoic membrane (CAM) grafting of adipose-derived stem cells (ADSCs) on plasma modified scaffolds for 7 days. A–D) Images of CAM-grafted scaffolds *in ovo* and E–H) after removal from the CAM. I–L). Scaffold sections stained with hematoxylin and eosin. Dotted lines indicate the edge of the scaffold material (s). Scale bar: 500 μm. M–P) Detection of laminin (green) and Q–T) vascular endothelial growth factor (VEGF, red) by immunocytochemistry in scaffold sections. Nuclei are in blue (Hoechst staining). Staining levels in POSS-PCU scaffolds appear to be higher in the Ar-modified scaffolds. U-V) Negative control where the primary antibody was omitted. Scale Bar 200 μm. POSS-PCU; Unmodified scaffolds, Ar: argon, N_2:_ nitrogen_,_ O_2:_ oxygen. (For interpretation of the references to colour in this figure legend, the reader is referred to the web version of this article.)

It was vital to assess if PSM scaffolds in this study could support angiogenesis and tissue ingrowth to ensure reliability and reproducibility and not only support differentiation. ADSC seeded in scaffold modified with Ar, N_2_ and O_2_ were CAM grafted and after 7 days gross morphology and expression of blood vessel markers was examined (Fig. 4). The H&E of the explanted CAMs demonstrated a similar structure after all plasma treatments with evidence of tissue growth (Fig. 4A–L). In addition, vessel formation was assessed in all the bioscaffolds by immunostaining for VEGF and laminin (Fig. 4M–V). Greater expression of these proteins was observed on Ar scaffolds than O_2_, N_2_ and control scaffolds with the CAM grafting model (Fig. 4).

## 4 Discussion

This study demonstrates that mRNA expression of osteogenic and chondrogenic differentiation of ADSCs can be enhanced using a very simple Ar surface functionalization technique. In addition, Ar modification enhanced ADSC adhesion to the 3D- polyurethane scaffold, ADSC proliferation and promoted tissue ingrowth in a CAM-grafting *in vivo* model.

The three gases examined in this study are the most commonly used gases utilised for PSM of biomaterials. Consistent with our study all forms of PSM increased stem cell adhesion and proliferation. Zanden *et al*. also demonstrated that Ar modification enhanced the embryonic stem cell (ESC) expansion compared to hydrogen and oxygen modification on polyurethane scaffolds [14]. Argon showed the optimal combination of surface functionality and roughness for cell expansion. Oxygen plasma modification showed the optimal cell adhesion for umbilical cord stem cells compared to argon on polyurethane polymers [15]. Oxygen plasma treatment has also increased the proliferation of mouse ESCs on polycaprolactone (PCL) nanofibres [16] and porcine MSCs on Poly-l-lactide (PLLA) nanofibres [17]. A combination of N_2_ and O_2_ plasma enhanced the proliferation of bone marrow stem cells on gelatin scaffolds due to the hydrophilic changes it imparted on the surface [18]. In this study, we illustrated that Ar promoted stem cell adhesion and proliferation better than N_2_ and O_2_ plasma. Cells interact with biomaterial surfaces by adhering to the proteins that are adsorbed onto the surface, which activates pathways to for cell adhesion. Cell attachment is affected by changes in hydrophobicity, charge, roughness and chemical composition [19]. FAK, encoded by the protein tyrosine kinase (PRK)-2 gene, is responsible for the recruitment of structural and signaling proteins to allow for cell adhesion [19]. Hence one reason why Ar may have promoted cell adhesion compared to N_2_ and O_2_ plasma is that the Ar surface was optimal for the recruitment of structural and signaling proteins for cell adhesion and consequently cell proliferation (Fig. 2).

The study also demonstrated that Ar enhanced osteogenesis and chondrogenesis by RT-qPCR (Fig. 3) and immunocytochemistry compared to N_2_ and oxygen O_2_. Argon plasma demonstrated an increase in aggrecan and collagen type II expression over 3 weeks in chondrogenic medium and similarly enhanced ALP, osteocalcin and collagen type I in osteogenic medium. To date only a few studies have compared different plasma gases to direct stem cell differentiation. Jahani *et al*. demonstrated that oxygen modified scaffolds may upregulate neuronal markers including Map-2 of MSCs on PCL fibres [20]. In agreement with our study, Zanden *et al*. demonstrated that Ar provided an optimal surface hydrophilicity and roughness compared to O_2_ plasma for stem cell expansion [15]. The authors demonstrate the Ar may provide an optimal surface topography compared to O_2_ plasma.

Numerous studies demonstrate that argon gas imparts a hydrophilic surface, by laying down hydroxyl, carbonyl and carboxyl chemical groups onto the surface and causes a minimal surface roughness [14,16,[21], [22], [23]]. This study further confirmed that for ADSCs expansion and differentiation Ar provides a surface with appropriate topography, hydrophilicity and roughness compared to N_2_ and O_2_ plasma. Our previous study demonstrated that the three gases caused very different effects on the surface chemistry and topography of the polyurethane scaffolds, which may explain our findings [11]. Argon plasma created a hydrophilic surface and etched the surface to become smoother with less contaminants than untreated polyurethane surfaces. On the other hand, O_2_ plasma modification caused a very hydrophilic surface, highly rough surface, elevated surface elastic modulus and very high levels of oxygen species deposition compared to untreated polyurethane surfaces [11]. Nitrogen caused a very mild effect on surface hydrophilicity, roughness and surface chemistry compared to Ar and O_2_ plasma. This demonstrates that Ar may provide the optimal hydrophilicity and topography that provides appropriate signals that induces osteogenesis and chondrogenesis whereas O_2_ modification is too intense and N_2_ is too mild. Argon is the most inert gas, which may explain some of these findings compared to N_2_ and O_2_ gas, which are more reactive [14]. However, future work should aim to identify the pathways that are modulated to direct the ADSCs towards osteogenic and chondrogenic differentiation. The enhanced adhesion and proliferation of the ADSCs on the Ar scaffold may also be accounted for by this data. It is likely that the interfacial changes to the material by Ar permitted the greatest number of proteins in an optimal conformation compared to other surface modifications [11].

In addition, to supporting chondrogenesis and osteogenesis scaffolds must be able to support angiogenesis to ensure the tissue *in vivo* survives. Several techniques have been investigated to enhance angiogenesis including modification of scaffold pore size and total porosity or implementing localized potent angiogenic growth factors [24,25]. The growth factor, VEGF has been widely reported as the key angiogenic factor for enhance vessel formation [24,25] and laminin is a key component of vessel walls. All scaffolds supported the ingrowth of tissue and vessel formation *in ovo* but those scaffolds treated with Ar promoted greater VEGF and laminin expression (Fig. 4). Several reports demonstrate that angiogenesis is affected by the surface topography of scaffolds but the mechanism by which this occurs is unclear. The surface properties induced by the Ar may have provided the optimal topography to allow for endothelial cell adhesion, proliferation, differentiation and consequently vessel formation *in vivo*.

The ability to control ADSC differentiation on biomaterial surface has been explored by the attachment of growth factors, extracellular matrix (ECM) proteins or by manipulating the scaffold surface architecture [26,27]. This study has shown that a simple surface modification with Ar plasma may promote osteogenic and chondrogenic differentiation of ADSCs. This is a simple one-process technique allowing easy translation to the clinical setting. We have also recently shown it can be effective in sterilizing polyurethane scaffolds [28]. This study highlighted that Ar modification can alter the chondrogenesis and osteogenesis phenotype but further investigation will understand how this may occur along the molecular pathway. In addition, long-term *in vivo* analysis should be performed to determine the efficacy and stability of the differentiated cells on the scaffolds with taking into consideration *in vivo* signals and cues.

## 5 Conclusions

Overall, this study suggests that hADSC-POSS-PCU scaffolds modified with Ar plasma modification provide appropriate surface cues to enhance differentiation of ADSCs towards bone and cartilage. Argon surface modification is easily transferrable to the clinical setting and suitable for modification of many biomaterial surfaces. Using argon modification for the generation of human bone and cartilage using adipose stem cells appears encouraging for skeletal tissue engineering applications.

## Appendix A Supplementary data

Supplementary Fig. 1. Biomaterial characteristics following argon (Ar), nitrogen (N_2_) and oxygen (O_2_) plasma surface modification (PSM). A) Contact angle measurements. Following 5 min of PSM treatment all surfaces became hydrophilic, with oxygen treated surfaces demonstrating the lowest contact angle. B) Surface Roughness. Following 5 min of PSM treatment oxygen surfaces showed an increase in roughness. C) Tensile Youngs Elastic Modulus. PSM did not affect the bulk mechanical properties of the material. D) Surface Morphology. Scanning Electron Microscopy Images demonstrate surface morphology following PSM varied depending on the gas utilised. a) Untreated control surfaces. b) Ar treated surfaces. c) N_2_ Treated surfaces d) O_2_ treated surfaces. Data summarized from [11]. Supplementary Fig. 2. Analysis of the extracellular matrix proteins secreted by the adipose-derived stem cells (ADSCs) on unmodified and plasma-modified scaffolds following osteogenic and chondrogenic differentiation. A–B) Alkaline phosphatase (ALP) activity and osteocalcin production following 14 and 21 days of osteogenic differentiation; both are enhanced on Ar-modified POSS-PCU scaffolds. C) Total collagen secretion after 21 days was greater on the Ar surfaces than other surfaces following osteogenic differentiation. D) Alizarin Red staining for osteogenic quantification; note significantly greater levels on the Ar scaffolds than on all other scaffolds (*p < 0.05. E–F) Elastin and glycosaminoglycans (GAG) detection following 21 days of chondrogenic differentiation; the higher levels are detected on Ar scaffolds (*p < 0.05). G) Alcian blue staining for chondrogenic quantification; note significantly greater expression on the Ar scaffolds than on all other scaffolds (*p < 0.05). Ar: argon: N_2:_ nitrogen; O_2:_ oxygen; Undiff: undifferentiated cells. Supplementary Fig. 3. Evaluation of osteogenic and chondrogenic differentiation on the plasma modified scaffolds. [A] Matrix deposition by adipose-derived stem cells (ADSCs) on plasma-modified scaffolds after 3 weeks of osteogenic differentiation. A–E) Alkaline phosphatase (ALP) expression detected by immunocytochemistry in ADSCs differentiated on unmodified (POSS-PCU), or argon- (Ar) (B), nitrogen- (N_2_)(D) and oxygen- (O_2_) (C) modified POSS-PCU and in undifferentiated ADSCs (Undiff) grown on POSS-PCU (A). Nuclei are in blue (Hoechst staining). Note that staining levels of both proteins appear to be greatest on Ar modified scaffolds (B, H). lower levels of expression are detected in undifferentiated ADSCs (E, K). Scale bar: 250 μm. [B] Matrix deposition by adipose-derived stem cells (ADSCs) on plasma scaffolds after 3 weeks of chondrogenic differentiation. A-E) Detection of Aggrecan detected by immunocytochemistry in ADSCs differentiated on unmodified (POSS-PCU), or argon- (Ar) (B), nitrogen- (N_2_) (C) and oxygen- (O_2_) (D) modified POSS-PCU and in undifferentiated ADSCs (Undiff) grown on POSS-PCU (A). Note that staining levels of both proteins appear to be greatest on Ar modified scaffolds (B). lower levels of expression are detected in undifferentiated controls (E). Nuclei are in blue (Hoechst staining). Scale Bar 250 μm. Image 1
