# Supplementary figures and images for: Argon plasma modification promotes adipose derived stem cells osteogenic and chondrogenic differentiation on nanocomposite polyurethane scaffolds; implications for skeletal tissue engineering
Source: Mater Sci Eng C Mater Biol Appl. 2019 Dec;105:110085. doi: 10.1016/j.msec.2019.110085 (PMC6892254; doi:10.1016/j.msec.2019.110085)

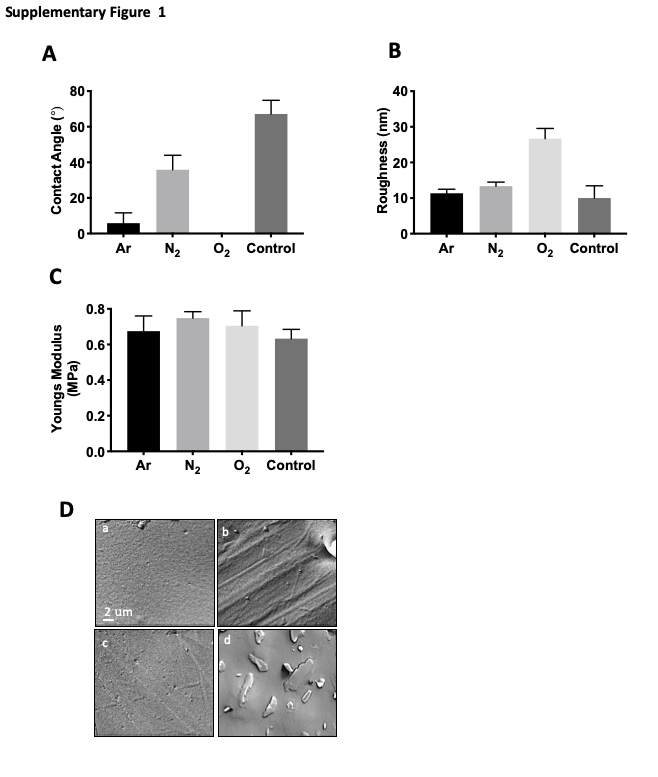


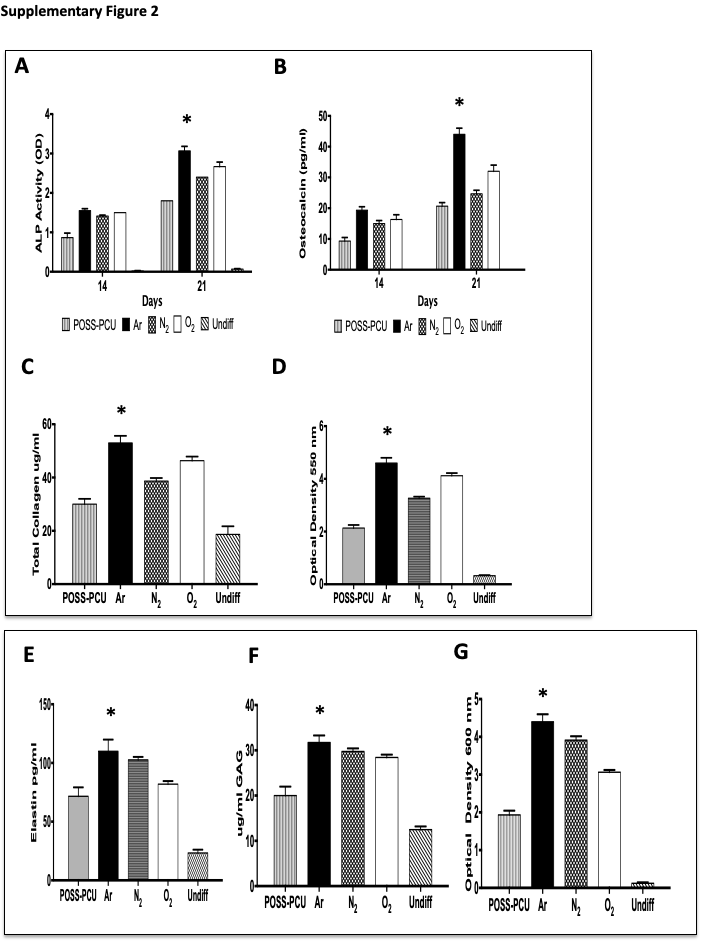


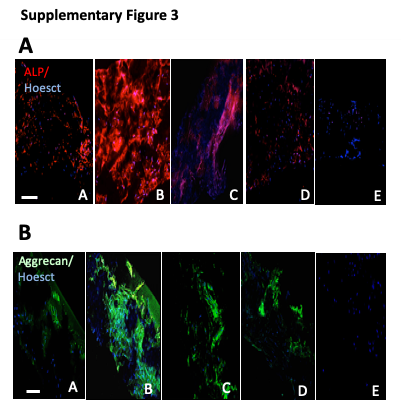

Supplement: Supplementary file 1 — Supplementary Fig. 1. Biomaterial characteristics following argon (Ar), nitrogen (N2) and oxygen (O2) plasma surface modification (PSM). A) Contact angle measurements. Following 5 min of PSM treatment all surfaces became hydrophilic, with oxygen treated surfaces demonstrating the lowest contact angle. B) Surface Roughness. Following 5 min of PSM treatment oxygen surfaces showed an increase in roughness. C) Tensile Youngs Elastic Modulus. PSM did not affect the bulk mechanical properties of the material. D) Surface Morphology. Scanning Electron Microscopy Images demonstrate surface morphology following PSM varied depending on the gas utilised. a) Untreated control surfaces. b) Ar treated surfaces. c) N2 Treated surfaces d) O2 treated surfaces. Data summarized from [11]. Supplementary Fig. 2. Analysis of the extracellular matrix proteins secreted by the adipose-derived stem cells (ADSCs) on unmodified and plasma-modified scaffolds following osteogenic and chondrogenic differentiation. A–B) Alkaline phosphatase (ALP) activity and osteocalcin production following 14 and 21 days of osteogenic differentiation; both are enhanced on Ar-modified POSS-PCU scaffolds. C) Total collagen secretion after 21 days was greater on the Ar surfaces than other surfaces following osteogenic differentiation. D) Alizarin Red staining for osteogenic quantification; note significantly greater levels on the Ar scaffolds than on all other scaffolds (*p < 0.05. E–F) Elastin and glycosaminoglycans (GAG) detection following 21 days of chondrogenic differentiation; the higher levels are detected on Ar scaffolds (*p < 0.05). G) Alcian blue staining for chondrogenic quantification; note significantly greater expression on the Ar scaffolds than on all other scaffolds (*p < 0.05). Ar: argon: N2: nitrogen; O2: oxygen; Undiff: undifferentiated cells. Supplementary Fig. 3. Evaluation of osteogenic and chondrogenic differentiation on the plasma modified scaffolds. [A] Matrix deposition by adipose-derived s [file mmc1.docx]
